# Mechanisms of mobile bearing dislocation in lateral unicompartmental knee replacement

**DOI:** 10.1177/09544119231195678

**Published:** 2023-09-30

**Authors:** Irene Yang, Greta Agustoni, David W Murray, Stephen J Mellon

**Affiliations:** Nuffield Department of Orthopaedics, Rheumatology and Musculoskeletal Sciences, University of Oxford, Oxford, UK

**Keywords:** Unicompartmental knee replacement, mobile bearing dislocation, lateral knee, mechanical rig, implant design

## Abstract

Mobile bearing dislocation occurs in 1– 6% of Oxford Domed Lateral replacements. Dislocations are predominantly medial, but can occur anteriorly or posteriorly. They tend to occur when the knee is flexed. It is not clear how dislocations can be prevented. A previously described mechanical rig for assessing mobile bearing dislocation was updated so as to study dislocation with the knee in flexion. Sub-categories for the description of each type of dislocation were introduced. Dislocation was only possible when the knee was distracted. As the amount of distraction possible in the knee is variable, the risk of dislocation is related to the amount of distraction in the rig necessary for a dislocation. The type of dislocation requiring the least distraction was medial `edge' dislocation in which the edge of the bearing dislocates onto the tibial wall, which is the most common type of dislocation. The amount of distraction necessary decreased the further the bearing was from the wall and with 50% posterior overhang. Rotation of the knee did not influence the amount of distraction. In conclusion dislocation can only occur if the lateral compartment is distracted. To reduce the dislocation risk, surgeons should aim to position the femoral and tibial components so that the bearing is as close as possible to the wall without jamming against it and the tibial component should be positioned flush with the posterior tibial cortex. If, during the surgery, the mobile bearing can easily be dislocated onto the wall the surgeon should consider changing to a fixed bearing. The tibial component should also be positioned flush with the posterior tibial cortex, as if it is too far forward this may contribute to dislocation.

## Graphical abstract

**Graphical abstract:**
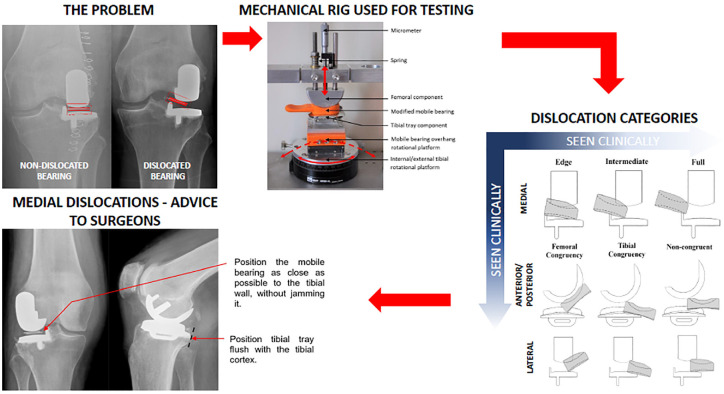

## Introduction

Isolated lateral compartment osteoarthritis occurs in about 10% of arthritic knees^1^ and can be effectively treated with Unicompartmental Knee Replacement (UKR). Lateral UKR accounts for 5%–10% of all UKR procedures.^2^ While implanted medially, the Oxford Knee has a well-documented history of clinical success,^3^ the lateral Oxford UKR has a relatively high incidence of mobile bearing dislocation compared to the medial. In the first published series^4^ that studied the original implant with a spherical femoral component, a flat tibial component and a mobile bearing with a concave superior (femoral component contacting) surface and a flat inferior (tibial component contacting) surface the dislocation incidence was 11%.^5^ For comparison, the medial Oxford UKR has a dislocation incidence of 0.6%.^6^ Many attempts have been made to address the problem of dislocation of the mobile bearing, including changes to the surgical technique such as introduction of a lateral parapatellar approach,^4,7^ internally rotating the tibial cut^8^ and avoiding excessive elevation of the lateral tibial joint line, which has been shown to increase the risk of dislocation.^9,10^ Surgeons have also attempted inserting screws into the tibial eminence, with the screw heads positioned above the tibial wall to augment the tibial wall height, to prevent recurrent medial dislocations.^8^ While some have reported success with this procedure,^8,11^ concern around potential metallosis resulting from metal-on-metal collision have also been expressed.^8^ Although these surgical technique changes appear to have decreased the dislocation rate appreciably, with one study reporting a decrease in the dislocation incidence from the initial 11% to 5%,^8^ the dislocation rate remained significantly higher than that of the medial Oxford UKR. In 2006, the design of the implant was changed and the Oxford Domed Lateral (ODL) UKR was introduced.^12^ The tibial component was given a convex tibial plateau while the mobile bearing was made biconcave to maintain congruency with the femoral and tibial components. The objective of these changes was to decrease dislocation incidence by increasing the mobile bearing ‘entrapment’ between the femoral and tibial components.^13^ A recent systematic review showed that introduction of the ODL reduced the mobile bearing dislocation incidence to 3.7%.^8^ If a dislocation occurs, probably the best treatment is conversion of the tibial component to a Fixed Lateral Oxford (FLO) tibial component. More importantly, if the dislocation rate could be substantially decreased, then mobile bearing lateral UKR would probably outperform fixed devices as they would not only provide better kinematics and function, but also they would have better long term implant survival. This is particularly pertinent given an ageing population and a greater demand for UKRs in younger and more active patient populations.^14^ Therefore, solutions to address the problem of mobile bearing dislocation are needed.

There have been few biomechanical studies of mobile bearing dislocation. For a dislocation to occur, the joint has to be distracted and the risk of dislocation is related to the amount of distraction necessary.^15^ In 2014 a study by Weston-Simons et al., used a custom-built mechanical rig to assess dislocation of the ODL implant mobile bearing.^13^ The rig featured a femoral (size medium) component, a tibial (size C) component and in between, a mobile bearing modified to include a handle. In the rig, the femoral component could be vertically distracted away from the tibial component. The tibial component was attached to a base stage capable of mediolateral (ML) translation, which could be controlled by a stage micrometer. Additionally, the tibial component could be rotated internally/externally. Using the rig and the mobile bearing with a handle, Weston-Simons et al.^13^ defined a set of component positions in which dislocations could occur. Further, each dislocation was also categorised broadly by the direction in which the mobile bearing could escape, either medially, laterally, anteriorly or posteriorly.

**Figure 1.**
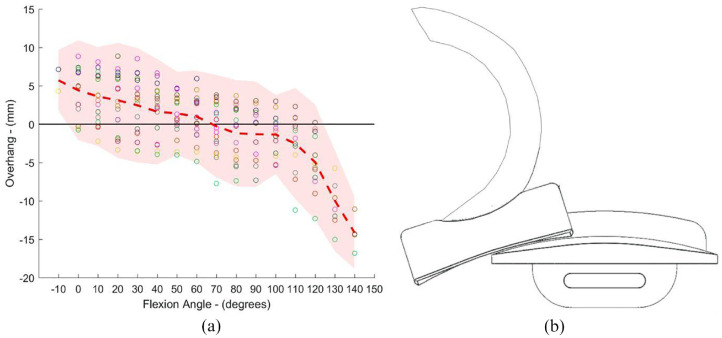
(a) Bearing overhang (mm) (relative to the tibial component) versus flexion angle (°) for the ODL^20^ and (b) Schematic depicting how knee flexion can result in up to 50% of the mobile bearing overhanging the tibial component posteriorly (relative to the right knee).

The study by Weston-Simons et al.,^13^ did not explore the relationship between between knee flexion and bearing dislocation, which is important as dislocations tend to occur in flexion. As the knee flexes, the femoral component rolls back relative to the tibial component^2^, causing the mobile bearing to overhang the back of the tibial component^20^ (Figure 1(a)). Martin et al.^16^ found that the amount of bearing overhanging the tibial component posteriorly can be up to 50% of the total bearing length when the knee is in high flexion (140°) (Figure 1(b)). In addition to bearing overhang, when the knee flexes, the tibial component internally rotates relative to the femoral component. Also, as there is more movement in the lateral as compared to the medial compartment of the knee,^17^ the mobile bearing in the lateral knee tends to rotate around the medial compartment on a curved track so the mobile bearing also moves mediolaterally.^13^

The aim of this study was to build on the previous work by Weston-Simons et al.^13^ and in particular, to study the effects of knee flexion, including overhang, rotation and mediolateral translation on dislocation. In addition to investigate dislocation in more detail, sub-categories for the description of each type of dislocation were added.

## Methods

### Updating the mechanical rig

**Figure 2.**
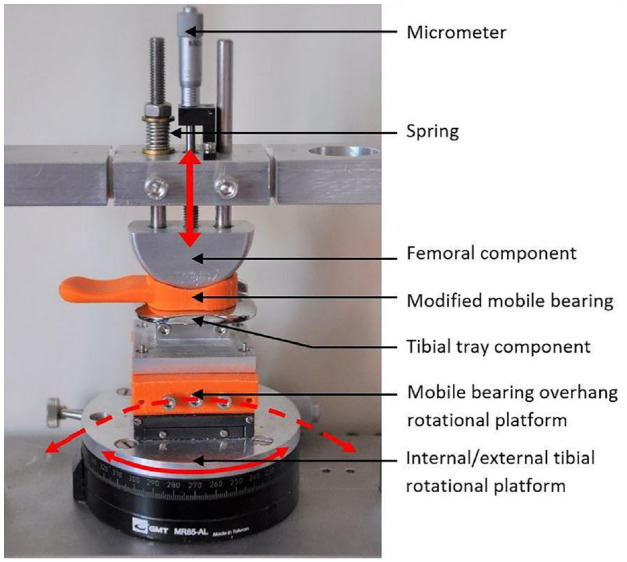
Updated mechanical rig for dislocation testing with the addition of a spring loaded micrometer (Mitutoyo Ltd.) for fine motion control, the fixture for the tibial stage and the modified bearing with a handle attached are shown in the picture. The components are depicted in the neutral position. Red arrows indicate the motion of the rig.

A previously described custom-built mechanical rig for mobile bearing dislocation assessment^13^ was updated to simulate knee flexion and bearing overhang (Figure 2). The original linear potentiometer was replaced with a micrometer (Mitutoyo Ltd.). The micrometer was accurate to 0.01 mm and facilitated simple and accurate distraction of the femoral component away from the tibial stage.

The tibial stage was modified to simulate bearing overhang, tibial rotation and mediolateral translation. To achieve this, a fixture for rotating the tibial component over a spherical of radius 75 mm was created to match the radius of curvature of the tibial dome surface. The fixture was drawn in SolidWorks (Dessault Systemes, MA, USA) and 3D printed. All 3D printed parts in this study were 3D printed in Polylactic Acid (PLA) using an Ultimaker 2 extended printer (Ultimaker B.V., The Netherlands).

**Figure 3.**
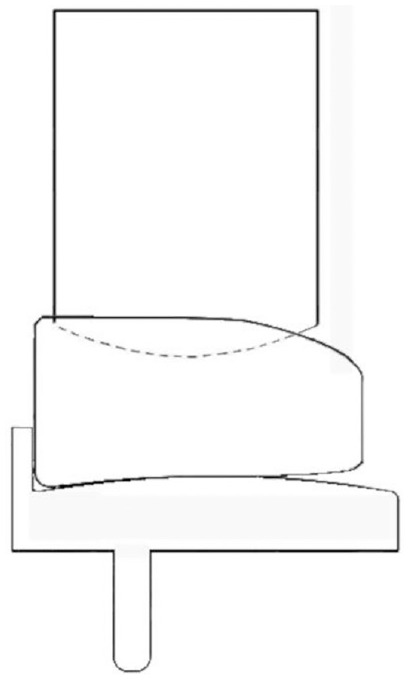
Image depicting 0° tibial rotation and 0 mm overhang: the starting position is shown with the mobile bearing flush against the wall of the tibial component and with the femoral component resting on the superior surface of the mobile bearing.

Based off the study by Martin et al.,^16^ the tibial component positioned at flexion angles of 0°, 90°, 130° and 140° were chosen for assessment. Therefore, at 0° of knee flexion, the mobile bearing was central on the tibial component (i.e. underhanging the posterior edge of the tibial component by 6.5 mm (see Figure 3). At flexion angles of 90°, 130° and 140° the mobile bearing was overhanging the tibial component posterior edge by 1, 10 and 17 mm, respectively (Table I). At 140°, the mobile bearing overhang of 17 mm, represented 50% of the mobile bearing AP length and was the maximum overhang observed *in vivo* in the Martin et al. study. To verify the tibial component was in the right location prior to testing, bearings with tabs corresponding to 1 mm, 10 mm and 17 mm of bearing overhang were drawn in SolidWorks and 3D printed.

**Table 1.** Modified mobile bearings designed. The mobile bearing without any tabs is the mobile bearing used for testing. The remaining bearings are used to setup overhang testing – red shows where the tabs were placed.

Amount of posterior mobile bearing overhang (mm)	Corresponding flexion angle (°)	Image
0	0	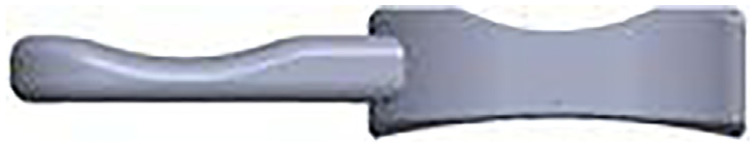
1	90	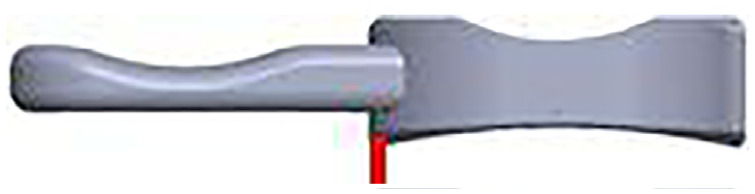
10	130	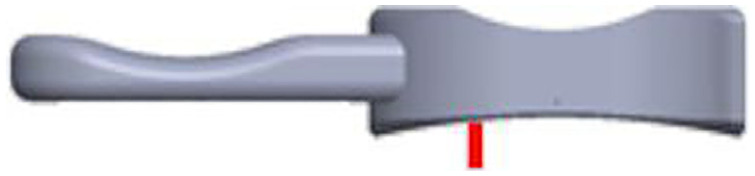
17	140	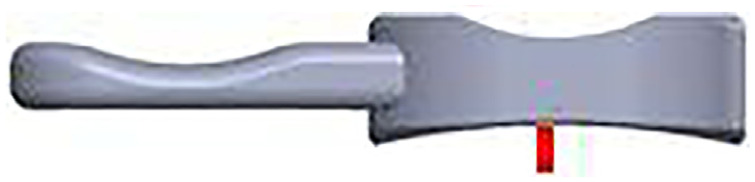

A size 3 C mobile bearing component (Zimmer Biomet Swindon, UK) was modified in SolidWorks and 3D printed to include a handle for testing.

### The effect of component positions on bearing dislocation

A total of 16,500 implant positions were tested. The testing parameters used for dislocation assessments are shown in Table 2.

**Table 2.** Dislocation testing parameters.

Testing parameter	Testing range (increments)/values
Bearing overhang values	−6.5 mm^†^, 1 mm, 10 mm, 17 mm
Rotational angles	−30°*, −10°*, 0°, 10°, 30°
Mediolateral translation (increment)	0.00–6.00 mm (0.25 mm)
Vertical distraction (increment)	0.00–8.00 mm (0.25 mm)

† Negative sign indicates underhang.

* Negative sign indicates external rotation.

The start position of testing was with the mobile bearing in contact with the tibial component, and positioned central on the tibia (no overhang) and with 0° rotation. Beginning with 0.00 mm ML translation (bearing flush against the wall of the tibial component (Figure 3), the femoral component was then distracted away from the mobile bearing, increasing the distance between the components from 0 mm (when the femoral component was in contact with the mobile bearing) up to a maximum of 8.00 mm in 0.25 mm increments. At each vertical distraction distance the mobile bearing was manipulated to see if it would dislocate either medially, laterally, anteriorly or posteriorly. To dislocate the mobile bearing, the minimum possible force was applied to ensure that dislocations were due to the geometric configuration, as opposed to the application of an external force. If a dislocation was possible, the vertical distraction at which the dislocation occurred, termed the Distraction to Dislocation (DD), was recorded. Testing was repeated for each ML translation distance (0.00 – 4.00 mm, 1 mm increments). To set up the next test, either the overhang value was changed, or the tibial rotation value.

The amount of vertical distraction required for a dislocation gives a quantity to the entrapment of the mobile bearing.

### Dislocation categories

**Figure 4.**
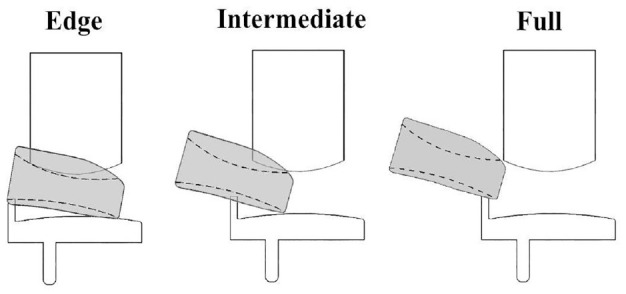
Schematic representing edge, intermediate and full medial dislocation with the Oxford domed lateral implant.

In addition to categorizing dislocations in the four directions as previously described by Weston-Simons et al.^13^ that is, medially over the tibial wall, anteriorly, posteriorly or laterally, sub-categories were further defined so that more detail about the dislocation type could be captured. A medial dislocation could be an ‘Edge’, ‘Intermediate’ or ‘Full’ dislocation (Figure 4).

**Figure 5.**
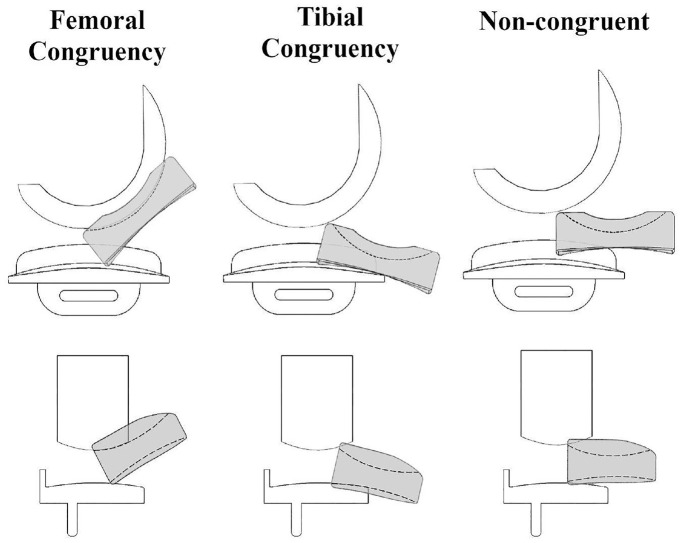
Schematic representing femoral congruency, tibial congruency or non-congruent dislocations applicable for anterior/posterior dislocations (top row) or lateral dislocations (bottom row) with the Oxford domed lateral implant.

Lateral, anterior and posterior dislocations were each also further divided into three sub-categories each to indicate if the mobile bearing was congruent with the femoral component (‘Femoral Congruency’), congruent with the tibial component (‘Tibial Congruency’) or not congruent with either (‘Non-congruent’) at the moment of dislocation (Figure 5).

If for any one configuration more than one dislocation category and/or sub-category was possible, the dislocation category plus sub-category that required the least DD was recorded. Dislocation occurrence, dislocation direction and the dislocation category were recorded in a spreadsheet in Excel (version 2016, Microsoft, Redmond, Washington, U.S.).

### Comparison with previous study

A comparison with the results from Weston-Simons et al.’s 2014 study^13^ was carried out. The positions tested are listed in Table 3. A total of 297 positions were compared.

**Table 3.** Weston-Simons et al.’s validation testing parameters.

Testing parameter	Testing range (increments)/values
Bearing overhang values	Implant is central (no bearing overhang)
Rotational angles	0°
Mediolateral translation (increment)	0.00–8.00 mm (1.00 mm increments)
Vertical distraction (increment)	0.00–8.00 mm (0.25 mm increments)

### Inter-observer assessment

The consistency in the dislocation data for two assessors (IY and GA) was assessed. GA repeated medial dislocation assessment using the modified mechanical rig, testing parameters listed in Table 4. GA tested a total of 561 implant positions.

**Table 4.** ICC Validation testing parameters.

Testing parameter	Testing range (increments)/values
Bearing overhang values	Implant is central (no bearing overhang)
Rotational angles	0°
Mediolateral translation (increment)	0.00–4.00 mm (0.25 mm increments)
Vertical distraction (increment)	0.00–8.00 mm (0.25 mm increments)

For each ML distance, the DD required for medial, lateral, anterior or posterior dislocation were statistically compared using an ICC: two-way random- effects model with type consistency and 95% Confidence Interval (CI) using IBM^®^ SPSS Statistical package (version 25) (SPSS Inc., Chicago, IL).

## Results

### The effect of component position on bearing dislocation

For all configurations, vertical distraction of the femoral component was required for mobile bearing dislocation to occur. For medial dislocation, the minimum DD was always via Edge dislocations and for anterior and lateral dislocations, the minimum DD was always through Femoral Congruency dislocations.

With the metal components at the starting position (bearing flush against the tibial wall, bearing central and without rotation), the amount of DD differed depending on the dislocation direction and the ML distance (Table 5). With 0.00 mm ML distance, the DD for a medial dislocation was 5.50 mm, 2.75 mm for a lateral, 5.75 mm for an anterior and 6.00 mm for a posterior dislocation. The DD for medial dislocations reduced to 2.25 mm with 6.00 mm ML distance, however, lateral, anterior or posterior dislocations were unaffected by changes to ML distance.

**Table 5.** The minimum DD (mm) required for medial, lateral, anterior or posterior dislocation of the mobile bearing given various configurations of the metal femoral and tibial components.

		Distraction to Dislocation (DD) (mm)							
		0.00 mm ML distance				6.00 mm ML distance			
Bearing overhang (mm)	Rotation angle (°)	Medial	Lateral	Anterior	Posterior	Medial	Lateral	Anterior	Posterior
−6.5†	0	5.50	2.75	5.75	6.00	2.50	2.75	5.75	6.00
	10	5.50	2.75	5.75	6.00	2.25	2.75	5.75	6.00
	−10*	5.50	2.75	5.75	6.00	2.25	2.75	5.75	6.00
	30	5.75	2.75	5.75	6.00	2.25	2.75	5.75	6.00
	−30*	5.50	2.75	5.75	6.00	2.25	2.75	5.75	6.00
1	0	5.50	2.75	5.75	6.00	2.25	2.75	5.75	6.00
	10	5.50	2.75	5.75	6.00	2.25	2.75	5.75	6.00
	−10*	5.50	2.75	5.75	6.00	2.00	2.75	5.75	6.00
	30	5.50	2.75	5.75	6.00	2.25	2.75	5.75	6.00
	−30*	5.50	2.75	5.75	6.00	2.00	2.75	5.75	6.00
10	0	5.25	2.75	5.75	6.00	2.00	2.75	5.75	6.00
	10	5.50	2.75	5.75	6.00	2.00	2.75	5.75	6.00
	−10*	5.50	2.75	5.75	6.00	2.00	2.75	5.75	6.00
	30	5.50	2.75	5.75	6.00	2.00	2.75	5.75	6.00
	−30*	5.50	2.75	5.75	6.00	2.00	2.75	5.75	6.00
17	0	4.50	3.00	5.75	6.00	0.75	2.75	5.75	6.00
	10	4.25	3.00	5.75	6.00	0.75	3.00	5.75	6.00
	−10*	4.25	3.00	6.00	6.00	1.00	3.00	6.00	6.00
	30	4.00	3.00	5.75	6.00	0.75	3.00	5.75	6.00
	−30*	4.25	3.00	6.00	6.00	0.75	3.00	6.00	6.00

† Negative sign indicates underhang.

* Negative sign indicates external rotation.

**Figure 6.**
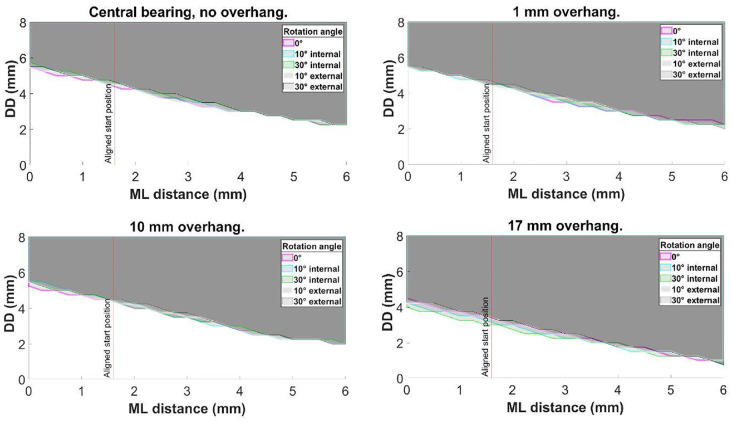
Central bearing with no overhang, and, with 1, 10 and 17 mm posterior bearing overhang: the effect of tibial rotation on medial dislocation.

Altering the tibial rotation angle from 0° to 10° and then 30° internal or external rotation had negligible effect on DD (Figure 6).

**Figure 7.**
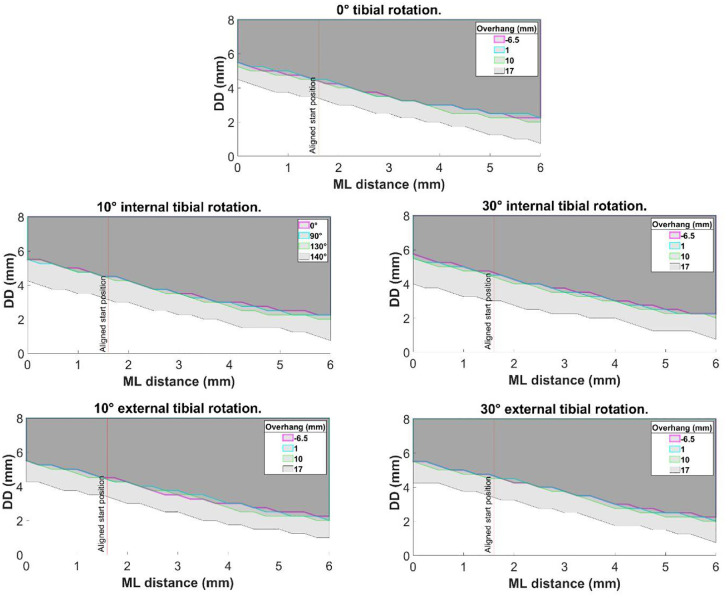
The effect of posterior bearing overhang on medial dislocation at 0°, 10° internal, 30° internal, 10° external and 30° external tibial rotation angles.

Increasing the amount of bearing overhang from central (6.50 mm underhang) to 1 mm or 10 mm posterior bearing overhang), also had negligible effect on the dislocation results, irrespective of the rotation angle (Figure 7). However, with 17 mm of overhang, the DD was reduced to 4.50 mm for a medial dislocation with 0 mm ML distance and 0.75 mm when the ML distance was increased to 6.00 mm (Figure 7). The DD remained 3.00 mm for a lateral dislocation and 5.75 mm for anterior and 6.00 mm for posterior dislocation.

### Comparison with previous study

For ML 0.00 – 8.00 mm, the medial DD was 6.40–5.00 mm for the previous study^13^ and 6.25–5.00 mm in this study (Table 6).

**Table 6.** Comparison of Weston-Simons et al., results with current study.

	Distraction to dislocation (mm)					
	Weston-Simons et al.’s^13^ study*			Current study		
Distance from the wall (mm)	Lateral	Medial	Anterior	Lateral	Medial	Anterior
0	5.10	6.40	8.20	4.00	6.25	7.00
1	5.10	5.90	8.10	4.00	6.00	7.00
2	5.00	5.50	8.00	4.00	5.50	7.00
3	5.10	5.80	8.00	4.00	5.75	7.00
4	5.10	5.40	8.00	4.00	5.50	7.00
5	5.00	5.40	8.10	4.00	5.75	7.00
6	5.10	5.30	8.10	4.00	5.75	7.00
7	5.10	5.40	8.00	3.75	5.75	7.00
8	4.90	5.00	7.88	3.75	5.00	7.00

* The standard deviations were ignored.

However, the lateral and anterior dislocation results reported in the current study were different to those reported previously.^13^ Lateral and anterior dislocation in this study required 4.00 mm and 7.00 mm DD respectively, compared with 5.00 and 8.00 mm in Weston-Simons et al. (Table 6).

### Inter-observer assessment

When comparing medial dislocation results between IY and GA for ML 0.00–4.00 mm, the medial DD was 5.50–3.00 mm for both observers. The statistical analysis showed excellent inter-observer reliability with an interclass correlation value of 0.995 (95%CI: 0.995–0.987) based on a mean rating (*k* = 2), consistency type assessment and two–way random effects model. Lateral dislocation showed complete agreement with a DD of 2.75 mm, whereas anterior/posterior dislocation showed a consistent offset of 1 mm (IY = 5.75 mm and GA = 6.75 mm).

## Discussion

In this study, a custom designed mechanical rig was used to study mobile bearing dislocations in the Oxford Domed Lateral in a series of physiologically relevant positions throughout the knee flexion range.

The study showed that with the ODL, distraction is essential for a bearing to dislocate. As the amount of distraction possible is different in different knees, DD is a measure of the risk of dislocation. The DD for medial dislocation was always lower than the DD for anterior and posterior dislocation explaining, in part, why medial dislocations are more common than anterior or posterior dislocations. Another reason is that for an anterior or posterior dislocation, the mobile bearing must be displaced half its length before it dislocates whereas for a medial dislocation it just needs to sublux a few millimetres onto the wall where it can become trapped. Medial dislocation risk depended on the relative ML position of the components, whereas the risk was independent of ML position for anterior or posterior dislocation. To minimise the risk of medial dislocation, the bearing should be as close as possible to the wall, but care should be taken to ensure it does not jam against the wall. Dislocations, irrespective of the direction, were unaffected by internal/external rotation or bearing overhang that occurs with knee flexion angles between 0° and 130°.

At 140° of knee flexion, the medial DD was reduced by 1 mm at all ML distances compared to the other flexion angles. The main barrier to medial dislocation is the tibial wall. The 1 mm reduction in DD was attributed to the reduction in apparent height of the wall at extreme bearing overhang, particularly since the posterior end of the wall is rounded. It must be noted, however, that 50% overhang was one extreme case^16^ and possibly the result of the tibial component being implanted too far forward resulting in excessive posterior bearing overhang with knee flexion. Instead, the tibial component should be positioned flush with the posterior tibia. Further, the relative increase in dislocation risk at 140° flexion is also likely to be countered by tension in the Lateral Collateral Ligament (LCL): evidence from an *in vivo* fluoroscopic study showed that the maximum laxity of the LCL is at 90° of flexion and at higher degrees of flexion it tightens.^18^

A Magnetic Resonance Imaging (MRI) study found that the lateral compartment of the knee distracted on average 6.7 ± 1.9 mm following application of a varus stress to a knee joint in 90° of knee flexion.^19^ Based on the results of the current study, this amount of distraction would permit a medial dislocation (Edge) and it is just enough for an anterior or posterior dislocation (Femoral congruency). Therefore, it is recommended that during the operation, once bone preparation, is complete, trial components should be inserted and the risk of dislocations assessed. If, with the leg in a figure of four position, the trial bearing can easily be dislocated medially, then a fixed rather than a mobile bearing device should be used. The FLO can be used interchangeably with the ODL. Therefore, without changing the bone preparation, the FLO should be implanted if there is a concern about medial bearing dislocation.

In this study lateral dislocations required the least amount of DD (2.75 mm), so it would be expected that this would be the most common type of dislocation. However, they do not occur clinically because the lateral compartment has a curtain of ligaments and other soft tissues which act as a physical barrier.^20^

In this study, the minimum DD for medial dislocation was always achieved through edge dislocations. In the comparison with the previous study,^13^ it was only possible to replicate the medial dislocation results previously reported if the specific manoeuvre described by Weston-Simons et al. was used, and the entire bearing could be removed in the medial direction (full dislocation). In other words, the medial dislocation results agreed only if DDs for Full medial dislocations were compared. This means that the results from the previous study were a subset of the results reported in this study. This suggests that expanding the definition of what constitutes a dislocation is beneficial to obtaining an accurate understanding of bearing dislocations.

### Study limitations

The main limitation of the study is that being a mechanical rig rather than a cadaver or clinical study, many factors, in particular those that might cause the mobile bearing to dislocate once the lateral compartment is distracted, cannot be assessed. Factors that might displace the bearing include soft tissue such as the popliteus tendon, bone, retained cement and perhaps synovial fluid. Factors relating to knee movement are also likely to be important. It is therefore recommended that cadaveric and dynamic studies are undertaken. However dislocation can only occur if there is knee distraction so the static rig studies are important. Further work should also look at the effect of relative varus/valgus alignment of the components, as surgical alignment may play a role in affecting the likelihood of dislocation.

## Conclusions

**Figure 8.**
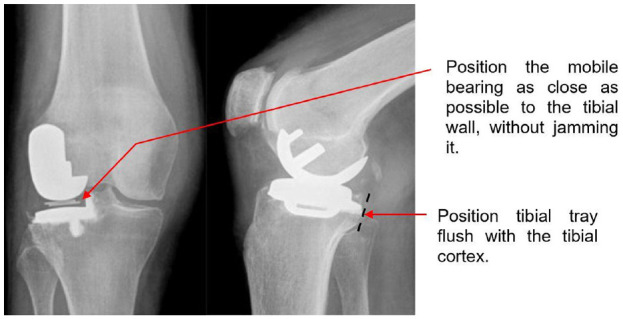
Figure adapted from Newman et al.^21^ to depict surgical recommendations.

In this study, newly established sub-categories for the description for medial, lateral and anterior/posterior dislocation were introduced. Using the new definitions, this study shows that distraction of the lateral compartment of the knee is essential for bearing dislocation to occur. Internal/external rotation or mobile bearing overhang do not increase the dislocation risk. The mobile bearings are more likely to dislocate medially than anteriorly or posteriorly because less distraction is needed for a medial dislocation. The further away the mobile bearing is from the wall of the tibial component, the greater the risk of medial dislocation. Therefore surgeons should aim to position the femoral and tibial components so that the mobile bearing is as close to the wall as possible (but does not jam against it) and the tibial component should also be positioned so that it is flush with the posterior tibial cortex, as if it is too far forward, then this might contribute to dislocation (Figure 8).

## References

[1] Plancher KD Alwine JT Petterson SC. Lateral unicondylar knee arthroplasty with lateral parapatellar incision returns athletes to moderate and vigorous sports: 2-11 year follow-up. Orthop J Sports Med. Epub ahead of print 31 July 2017. DOI: 10.1177/2325967117S00292.

[2] van der List JP Chawla H Zuiderbaan HA , et al. Patients with isolated lateral osteoarthritis: unicompartmental or total knee arthroplasty? Knee 2016; 23(6): 968–974. 27810429 10.1016/j.knee.2016.06.007

[3] Pandit H Jenkins C Gill HS , et al. Minimally invasive Oxford phase 3 unicompartmental knee replacement: results of 1000 cases. J Bone Joint Surg Br 2011; 93(2): 198–204. 21282759 10.1302/0301-620X.93B2.25767

[4] Gunther TV Murray DW Miller R , et al. Lateral unicompartmental arthroplasty with the Oxford meniscal knee. Knee 1996; 3(1–2): 33–39.

[5] Goodfellow JW O’Connor J. Clinical results of the Oxford knee. Surface arthroplasty of the tibiofemoral joint with a meniscal bearing prosthesis. Clin Orthop Relat Res 1986; 205: 21–42. 3698380

[6] Mohammad HR Strickland L Hamilton TW , et al. Long-term outcomes of over 8,000 medial Oxford phase 3 unicompartmental knees-a systematic review. Acta Orthop 2018; 89(1): 101–107. 28831821 10.1080/17453674.2017.1367577 PMC5810816

[7] Goodfellow JW Kershaw CJ Benson MK , et al. The Oxford knee for unicompartmental osteoarthritis. The first 103 cases. J Bone Joint Surg Br 1988; 70(5): 692–701. 3192563 10.1302/0301-620X.70B5.3192563

[8] Pandit H Jenkins C Beard DJ , et al. Mobile bearing dislocation in lateral unicompartmental knee replacement. Knee 2010; 17(6): 392–397. 19919897 10.1016/j.knee.2009.10.007

[9] Robinson BJ Rees JL Price AJ , et al. Dislocation of the bearing of the Oxford lateral unicompartmental arthroplasty. A radiological assessment. J Bone Joint Surg Br 2002; 84(5): 653–657. 12188479 10.1302/0301-620x.84b5.12950

[10] Streit MR Walker T Merle C , et al. Mobile-bearing lateral unicompartmental knee replacement with the Oxford domed tibial component: an independent series. J Bone Joint Surg Br 2012; 94(10): 1356–1361. 23015560 10.1302/0301-620X.94B10.29119

[11] Schelfaut S Beckers L Verdonk P , et al. The risk of bearing dislocation in lateral unicompartmental knee arthroplasty using a mobile biconcave design. Knee Surg Sports Traumatol Arthrosc 2013; 21(11): 2487–2494. 22926671 10.1007/s00167-012-2171-7

[12] Bare JV Gill HS Beard DJ , et al. A convex lateral tibial plateau for knee replacement. Knee 2006; 13(2): 122–126. 16403637 10.1016/j.knee.2005.09.001

[13] Weston-Simons JS Kendrick BJL Mentink MJA , et al. An analysis of dislocation of the domed Oxford lateral unicompartmental knee replacement. Knee 2014; 21(1): 304–309. 23673196 10.1016/j.knee.2013.04.008

[14] Altuntas AO Alsop H Cobb JP. Early results of a domed tibia, mobile bearing lateral unicompartmental knee arthroplasty from an independent centre. Knee 2013; 20(6): 466–470. 23274066 10.1016/j.knee.2012.11.008

[15] Yang I Gammell JD Murray DW , et al. Application of a robotics path planning algorithm to assess the risk of mobile bearing dislocation in lateral unicompartmental knee replacement. Sci Rep 2022; 12: 2068. 35136092 10.1038/s41598-022-05938-w PMC8825833

[16] Martin BR Pegg EC van Duren BH , et al. Posterior bearing overhang following medial and lateral mobile bearing unicompartmental knee replacements. J Orthop Res 2019; 37(9): 1938–1945. 31058359 10.1002/jor.24339

[17] Iwaki H Pinskerova V Freeman MAR . Tibiofemoral movement 1: the shapes and relative movements of the femur and tibia in the unloaded cadaver knee. J Bone Joint Surg Br 2000; 82(8): 1189–1195. 11132285 10.1302/0301-620x.82b8.10717

[18] Hosseini A Qi W Tsai TY , et al. In vivo length change patterns of the medial and lateral collateral ligaments along the flexion path of the knee. Knee Surg Sports Traumatol Arthrosc 2015; 23(10): 3055–3061. 25239504 10.1007/s00167-014-3306-9 PMC4368498

[19] Tokuhara Y Kadoya Y Nakagawa S , et al. The flexion gap in normal knees. An MRI study. The J Bone Joint Surg Br 2004; 86(8): 1133–1136. 15568525 10.1302/0301-620x.86b8.15246

[20] Seebacher JR Inglis A Marshall JL , et al. The structure of the posterolateral aspect of the knee. J Bone Joint Surg Am 1982; 64(4): 536–541. 7068696

[21] Newman SDS Alsop H Cobb JP , et al. Up to 10 year follow-up of the Oxford domed lateral partial knee replacement from an independent centre. Knee 2017; 24(6): 1414–1421. 28974402 10.1016/j.knee.2017.05.001

